# A colorimetric loop-mediated isothermal amplification assay (c-LAMP) for rapid detection of *Pythium insidiosum*

**DOI:** 10.1016/j.heliyon.2024.e40478

**Published:** 2024-11-17

**Authors:** Thanawat Sridapan, Chalisa Jaturapaktrarak, Thidarat Rujirawat, Atisak Jiaranaikulwanich, Chompoonek Yurayart, Theerapong Krajaejun

**Affiliations:** a Department of Pathology, Faculty of Medicine, Ramathibodi Hospital, Mahidol University, Bangkok, 10400, Thailand; b Research Center, Faculty of Medicine, Ramathibodi Hospital, Mahidol University, Bangkok, 10400, Thailand; c Department of Microbiology and Immunology, Faculty of Veterinary Medicine, Kasetsart University, Bangkok, 10900, Thailand

**Keywords:** *Pythium insidiosum*, Pythiosis, Diagnosis, LAMP, Hydroxynaphthol blue

## Abstract

Pythiosis, caused by *Pythium insidiosum*, is a severe infectious disease affecting humans and animals worldwide. There is an urgent need for a simple and rapid detection method for pythiosis, especially in remote areas where this disease is prevalent. To address this, a colorimetric loop-mediated isothermal amplification assay (c-LAMP) using hydroxynaphthol blue dye as a color indicator has been developed. This method utilized a one-step closed-tube system under a single temperature reaction to detect *P. insidiosum*, minimizing DNA carry-over contamination and eliminating the need for expensive tools. The test result can be easily read through the color change from violet (negative) to sky blue (positive). When tested with DNA samples from *P. insidiosum* (*n* = 51) and other fungi (*n* = 70), c-LAMP showed a detection sensitivity, specificity, and accuracy of 100.0 %, 95.7 %, and 97.5 %, respectively. The assay detection limit was 1 x 10^−5^ ng of DNA template, 10,000 times lower than the reference multiplex PCR assay (m-PCR). c-LAMP also showed a faster assay turnaround time, taking only 65 min, as opposed to the 180 min required for m-PCR. This newly established c-LAMP is rapid, cost-effective, and efficient, making it a promising tool for detecting *P. insidiosum* in resource-limited laboratories.

## 1 Introduction

Pythiosis is caused by *Pythium insidiosum*, an aquatic oomycete known to infect humans and various animals living in subtropical and tropical areas [1]. The clinical signs of pythiosis can vary, including cutaneous/subcutaneous, vascular, ocular, gastrointestinal, and disseminated types of infection [2]. The disease is difficult to treat due to the limited response to antifungal medicines. Complete surgical excision of the infected tissue is the primary and effective treatment option [3,4]. The high morbidity and mortality of pythiosis could be a consequence of the lack of an accurate and timely diagnosis [5]. Early diagnosis of *P. insidiosum* is essential to improve the clinical outcome of the affected patients. The traditional microbiological method that relies on culturing to isolate the causative agent of infection is both labor-intensive and time-consuming. Additionally, it often fails to identify *P. insidiosum* from clinical specimens [1,6]. While immunodiagnostic assays, such as immunochromatographic tests, have been developed to detect antibodies against *P. insidiosum*, these tests can show cross-reactivity with antibodies against other fungi, compromising their specificity [[5], [6], [7], [8]]. In contrast, nucleic acid-based tests (NATs), particularly polymerase chain reaction (PCR), have gained popularity due to their high detection efficiency, increased throughput, and shorter turnaround times for diagnosing *P. insidiosum* infections [5,6,9].

Several PCR-based assays have been developed for detecting *P. insidiosum* [6]. These molecular techniques are more efficient compared to other methods, such as morphological identification and immunodiagnostic tests [6,10,11]. However, a significant drawback of PCR-based assays is the need for a thermocycler to amplify DNA [6,11,12], which might not be available in resource-limited laboratories. In light of this, the isothermal amplification approach, specifically loop-mediated isothermal amplification (LAMP), has emerged as an alternative technique to amplify a target sequence at a constant temperature without requiring a thermocycler [13]. LAMP relies on the *Bst* DNA polymerase for auto-cycling strand displacement DNA synthesis under an isothermal condition ranging from 60 to 65 °C [14]. This technique has gained attention due to its simplicity, speed, specificity, and cost-effectiveness, making it suitable for on-site diagnostic applications [13,15,16]. LAMP has been increasingly used for rapid detection of various microorganisms, including *P. insidiosum* [14,16,17]. Initially, the detection of LAMP products relied on identifying a ladder-like band pattern through gel electrophoresis, potentially leading to aerosol DNA carry-over contamination and false-positive results due to opened-tube procedures and sample pipetting [16,17]. Therefore, a closed-tube end-point detection system for the LAMP products is essential. While the addition of fluorescent dyes has led to the development of closed-system real-time fluorescence devices for confirming LAMP reactions [[18], [19], [20], [21]], this technology often necessitates the use of expensive reagents (such as fluorescent dyes) and equipment (such as fluorescent signal detectors), which can limit its widespread adoption.

Colorimetric methods have been used to visually detect LAMP products and make it convenient to interpret results [16]. Some researchers have employed DNA intercalating dyes, such as SYBR green I, for end-point detection of LAMP products. In this method, only the positive samples turn from orange to green [17,[22], [23], [24]]. However, because SYBR green I inhibits the amplification process, it is added at the end of the LAMP reaction [25], which may lead to DNA carry-over contamination. Alternatively, an end-point colorimetric detection of LAMP products using a metal ion indicator, such as hydroxynaphthol blue (HNB) dye premixed in the amplification reaction as a closed-tube system, has been described [[25], [26], [27], [28], [29], [30], [31], [32]]. Magnesium ions (Mg^2+^) are released from HNB to become insoluble magnesium pyrophosphate when dNTPs are incorporated into the amplified DNAs. In a positive amplification reaction, HNB lacking Mg^2+^ ions turns the reaction solution from violet to sky blue, allowing a one-step closed-tube procedure with instant visual identification of the LAMP product [25]. This study aims to develop a colorimetric LAMP assay (c-LAMP) for detecting *P. insidiosum*. The assay is simple, efficient, and cost-effective for diagnosing pythiosis, especially in a resource-limited clinical laboratory.

## 2 Materials and methods

### 2.1 Microorganisms and DNA preparations

Fifty-one *P. insidiosum* isolates from humans, animals, and the environment, and 70 clinically relevant fungi were recruited to evaluate c-LAMP in comparison with the reference multiplex PCR assay (m-PCR) [17,33] (Table 1). Genomic DNA was extracted from mycelium using established mechanical disruption methods, which involved grinding the organism in liquid nitrogen, as described previously [34,35]. The DNA concentration was accessed for each sample using a Qubit 3 Fluorometer [36] and Nanodrop spectrophotometry (Thermo Fisher Scientific), while the DNA purity was determined using the A260/A280 ratio. The extracted DNA samples were stored at −20 °C until used.

**Table 1:** List of *P. insidiosum* (*n* = 51) and control fungi (*n* = 70) used for the evaluation of c-LAMP and m-PCR.

No.	Organisms	Strain ID	Genotypes	Result interpretation	
				m-PCR	c-LAMP
1	*Pythium insidiosum*	CBS 578.85	Clade I	**+**	**+**
2	*Pythium insidiosum*	CBS 579.85	Clade I	**+**	**+**
3	*Pythium insidiosum*	CBS 577.85	Clade I	**+**	**+**
4	*Pythium insidiosum*	CBS 573.85	Clade I	**+**	**+**
5	*Pythium insidiosum*	ATCC200269	Clade I	**+**	**+**
6	*Pythium insidiosum*	EQ 02	Clade I	**+**	**+**
7	*Pythium insidiosum*	EQ 04	Clade I	**+**	**+**
8	*Pythium insidiosum*	EQ 05	Clade I	**+**	**+**
9	*Pythium insidiosum*	EQ 06	Clade I	**+**	**+**
10	*Pythium insidiosum*	EQ 09	Clade I	**+**	**+**
11	*Pythium insidiosum*	EQ 10	Clade I	**+**	**+**
12	*Pythium insidiosum*	EQ 12	Clade I	**+**	**+**
13	*Pythium insidiosum*	EQ 13	Clade I	**+**	**+**
14	*Pythium insidiosum*	P45_Br	Clade I	**+**	**+**
15	*Pythium insidiosum*	P03	Clade II	**+**	**+**
16	*Pythium insidiosum*	P16	Clade II	**+**	**+**
17	*Pythium insidiosum*	P23	Clade II	**+**	**+**
18	*Pythium insidiosum*	P34	Clade II	**+**	**+**
19	*Pythium insidiosum*	P36	Clade II	**+**	**+**
20	*Pythium insidiosum*	P37	Clade II	**+**	**+**
21	*Pythium insidiosum*	Pi-S	Clade II	**+**	**+**
22	*Pythium insidiosum*	ATCC64221	Clade II	**+**	**+**
23	*Pythium insidiosum*	CBS 101039	Clade II	**+**	**+**
24	*Pythium insidiosum*	CM07	Clade II	**+**	**+**
25	*Pythium insidiosum*	P38	Clade II	**+**	**+**
26	*Pythium insidiosum*	P39	Clade II	**+**	**+**
27	*Pythium insidiosum*	P40	Clade II	**+**	**+**
28	*Pythium insidiosum*	P41	Clade II	**+**	**+**
29	*Pythium insidiosum*	P50	Clade II	**+**	**+**
30	*Pythium insidiosum*	KCB06	Clade II	**+**	**+**
31	*Pythium insidiosum*	KCB07	Clade II	**+**	**+**
32	*Pythium insidiosum*	RM902	Clade II	**+**	**+**
33	*Pythium insidiosum*	P42	Clade II	+	+
34	*Pythium insidiosum*	RT01	Clade II	+	+
35	*Pythium insidiosum*	RT02	Clade II	+	+
36	*Pythium insidiosum*	44P21-2	Clade II	+	+
37	*Pythium insidiosum*	59P21-2	Clade II	+	+
38	*Pythium insidiosum*	MCC17	Clade III	+	+
39	*Pythium insidiosum*	MCC13	Clade III	+	+
40	*Pythium insidiosum*	P13	Clade III	+	+
41	*Pythium insidiosum*	P21	Clade III	+	+
42	*Pythium insidiosum*	P26	Clade III	+	+
43	*Pythium insidiosum*	P33	Clade III	+	+
44	*Pythium insidiosum*	ATCC90586	Clade III	+	+
45	*Pythium insidiosum*	P43	Clade III	+	+
46	*Pythium insidiosum*	P46	Clade III	+	+
47	*Pythium insidiosum*	P48	Clade III	+	+
48	*Pythium insidiosum*	P49	Clade III	+	+
49	*Pythium insidiosum*	KCB03	Clade III	+	+
50	*Pythium insidiosum*	KCB08	Clade III	+	+
51	*Pythium insidiosum*	KCB09	Clade III	+	+
52	*Pythium aphanidermatum*	ATCC32230	NA	–	+
53	*Pythium catenulatum*	RM9-06	NA	–	+
54	*Pythium rhizo-oryzae*	RCB01	NA	–	+
55	*Acremonium* spp.	RA012	NA	–	–
56	*Alternaria* spp.	RA061	NA	–	–
57	*Alternaria* spp.	RA121	NA	–	–
58	*Alternaria* spp.	RA149	NA	–	–
59	*Aspergillus* spp.	RA081	NA	–	–
60	*Aspergillus* spp.	RA099	NA	–	–
61	*Aspergillus* spp.	RA100	NA	–	–
62	*Aspergillus* spp.	RA114	NA	–	–
63	*Aspergillus* spp.	RA122	NA	–	–
64	*Aspergillus* spp.	RA123	NA	–	–
65	*Aspergillus fumigatus*	RA018	NA	–	–
66	*Aspergillus fumigatus*	RA020	NA	–	–
67	*Aspergillus fumigatus*	RA075	NA	–	–
68	*Aspergillus flavus*	RA115	NA	–	–
69	*Aspergillus flavus*	RA132	NA	–	–
70	*Aspergillus flavus*	RA139	NA	–	–
71	*Aspergillus glaucus*	RA071	NA	–	–
72	*Aspergillus niger*	RA021	NA	–	–
73	*Aspergillus terreus*	RA097	NA	–	–
74	*Aspergillus terreus*	RA109	NA	–	–
75	*Chaetomium* spp.	RA028	NA	–	–
76	*Chaetomium* spp.	RA134	NA	–	–
77	*Chrysosporium* spp.	RA024	NA	–	–
78	*Cladophialophora bantiana*	RA037	NA	–	–
79	*Cladosporium* spp.	RA026	NA	–	–
80	*Cladosporium* spp.	RA120	NA	–	–
81	*Cladosporium* spp.	RA133	NA	–	–
82	*Cladosporium* spp.	RA138	NA	–	–
83	*Curvularia* spp.	RA153	NA	–	–
84	*Curvularia* spp.	RA070	NA	–	–
85	*Curvularia* spp.	RA098	NA	–	–
86	*Curvularia* spp.	RA110	NA	–	–
87	*Curvularia* spp.	RA118	NA	–	–
88	*Curvularia* spp.	RA143	NA	–	–
89	*Curvularia* spp.	RA124	NA	–	–
90	*Curvularia* spp.	RA130	NA	–	–
91	*Curvularia* spp.	RA136	NA	–	–
92	*Curvularia* spp.	RA137	NA	–	–
93	*Daldinia* spp.	RA027	NA	–	–
94	*Exerohilum rostratum*	RA131	NA	–	–
95	*Exophiala* spp.	RA023	NA	–	–
96	*Fusarium* spp.	RA013	NA	–	–
97	*Fusarium* spp.	RA015	NA	–	–
98	*Fusarium* spp.	RA042	NA	–	–
99	*Fusarium* spp.	RA135	NA	–	–
100	*Histoplasma capsulatum*	RA072	NA	–	–
101	*Microsporum gypseum*	RA038	NA	–	–
102	*Neoscytalidium* spp.	RA040	NA	–	–
103	Non-sporulating fungi	RA101	NA	–	–
104	*Paecilomyces variotii*	RA141	NA	–	–
105	*Penicillium* spp.	RA036	NA	–	–
106	*Rhizopus* spp.	RA065	NA	–	–
107	*Scedosporium apiospermum*	RA150	NA	**-**	**-**
108	*Syncephalastrum* spp.	RA041	NA	**-**	**-**
109	*Syncephalastrum* spp.	RA145	NA	**-**	**-**
110	*Talaromyces marneffei*	RA060	NA	**-**	**-**
111	*Talaromyces marneffei*	RA083	NA	**-**	**-**
112	*Talaromyces marneffei*	RA105	NA	**-**	**-**
113	*Talaromyces marneffei*	RA106	NA	**-**	**-**
114	*Talaromyces marneffei*	RA142	NA	**-**	**-**
115	*Trichoderma* spp.	RA066	NA	**-**	**-**
116	*Trichoderma* spp.	RA126	NA	**-**	**-**
117	*Trichophyton* spp.	RA111	NA	**-**	**-**
118	*Trichophyton mentagrophytes*	RA039	NA	**-**	**-**
119	*Trichophyton mentagrophytes*	RA104	NA	**-**	**-**
120	*Trichophyton rubrum*	RA077	NA	**-**	**-**
121	*Trichophyton rubrum*	RA128	NA	**-**	**-**

Footnote.

‘+’ indicates a positive amplification reaction.

‘–’ indicates a negative amplification reaction.

### 2.2 LAMP primers

The following set of LAMP primers targets the ribosomal DNA internal transcribed spacer (ITS) of *P. insidiosum*. The set includes 4 previously described primers [17]: 2 outer primers (F3: 5′-GGCAGAATGTGAGGTGTCTC-3'; and B3: 5′-GGAAACAACACCCCGTCAG-3′) and 2 inner primers (FIP: 5′-ACAGATCACTGCGTTCGAGCAT-TTTT-GGAGATAGCACGAGTCCCT-3'; and BIP: 5′-TCAGATTGCTTTGCGCTGGTGG-TTTT-CCGAAGCCTAACATACCGC-3′). Additionally, a forward loop primer (LF: 5′-GACACAAGAGAGATCAACGTACATT-3′) and a backward loop primer (LB: 5′-AGGACATTAAGGAGATGACCTCTAT-3′) were newly designed using Primer Explorer software version 5 (http://primerexplorer.jp/lampv5e/index.html). All 6 LAMP primers were synthesized and then purified to high-affinity purification (HAP) grade by Bio Basic Inc., Canada.

### 2.3 Colorimetric LAMP reaction

The regular LAMP reaction was carried out in a 25 μl mixture using a set of 4 primers (F3, B3, FIP, and BIP) as previously described [17], and in another set using a total of 6 primers by including 2 additional primers (LF and LB). Representative DNA samples from *P. insidiosum* (*n* = 4) and various fungi (*n* = 5) were used for assay optimization. HNB was included in the reaction mixture. In the modified protocol, each reaction contained a mixture of 1 × ThermoPol reaction buffer, 6 mM MgSO_4_, 8 U of *Bst* DNA polymerase, large fragment (New England Biolabs Inc., USA), 1.4 mM of dNTPs (New England Biolabs Inc., USA), 0.4 M betaine (Sigma, USA), 0.2 μM F3 and B3 primers, 1.6 μM FIP and BIP primers, 0.4 μM LF and LB primers, 0.12 mM HNB (3 mM, Loba Chemie, India) and 2 μl of template DNA (∼50 ng/μl). The blank control reaction contained no template DNA. The reaction was conducted in a Mastercycler Nexus Gradient thermocycler at 65 °C for 60 min, followed by heat inactivation at 85 °C for 5 min. A color change from violet to sky blue in the reaction tube indicated a “positive” result, while no color change (violet) indicated a “negative” result. The presence of LAMP products was confirmed by 1.5 % agarose gel electrophoresis analysis, stained with SERVA DNA stain G (SERVA Electrophoresis GmbH, Germany), and photographed under UV light using a UV transilluminator (Bio-Rad, USA).

### 2.4 Multiplex PCR analysis

m-PCR was conducted following a previously reported protocol [17,33]. Each 25 μl reaction consisted of 1 × *Taq* buffer with KCl, 2 mM of MgCl_2_, 0.2 mM of dNTPs, 0.75 unit of *Taq* DNA polymerase (Thermo Fisher Scientific, USA), 0.12 μM of the forward primer ITS1 (5′-TCCGTAGGTGAACCTGCGG-3′), 0.07 μM each of the reverse primers R1 (5′-CCTCACATTCTGCCATCTCG-3′), R2 (5′-ATACCGCCAATAGAGGTCAT-3′), and R3 (5′-TTACCCGAAGGCGTCAAAGA-3′), and 2 μl of template DNA (∼50 ng/μl). The amplification condition was set as follows: initial denaturation at 95 °C for 5 min, followed by 30 cycles of 95 °C for 30 s, 59 °C for 30 s, and 72 °C for 45 s, and a single final extension at 72 °C for 10 min, using a Mastercycler Nexus Gradient thermocycler (Eppendorf, Germany). The m-PCR products were analyzed using 1.5 % agarose gel electrophoresis and stained with SERVA DNA Stain G.

### 2.5 Limit of detection of c-LAMP and m-PCR

The limit of detection (LOD) for c-LAMP and m-PCR assays was determined by conducting 10-fold serial dilutions (starting from 10 ng of total template DNA) of 3 reference *P. insidiosum* strains CBS573.85 (genotype: clade I), Pi-S (clade II), and MCC13 (clade III). As a result, the template DNAs calculatedly ranged from 10 to 1 x 10^−8^ ng per reaction. The LOD was defined as the lowest concentration of template DNA that still produced a positive result. Each reaction was conducted 3 times, and 2 independent researchers confirmed the results.

### 2.6 Performance evaluation of c-LAMP

The diagnostic performance of c-LAMP, in comparison with m-PCR, was assessed using DNA samples from 51 *P. insidiosum* isolates and 70 control fungi. The HNB colorimetric method was used to check the presence of an amplicon only for c-LAMP, while agarose gel electrophoresis was used to determine the results for both assays. Each experiment was conducted twice. Detection sensitivity, specificity, and accuracy of c-LAMP and m-PCR assays were calculated using MedCalc statistical software (https://www.medcalc.org/calc/diagnostic_test.php) and reported with 95 % confidence intervals (CI).

To investigate a false-positive result, the chemical reagents and related supplies were replaced with a new clean set, and the working space was decontaminated before c-LAMP was performed using the sample prepared from an organism in question. Additionally, primer cross-annealing was assessed by aligning the c-LAMP primers with their corresponding target ITS sequences from *P. insidiosum* and selected organisms using the Clustal W package in Bioedit software version 7.2.5 [37] to check for any sequence mismatches.

## 3 Results

### 3.1 Optimization of c-LAMP

**Fig. 1:**
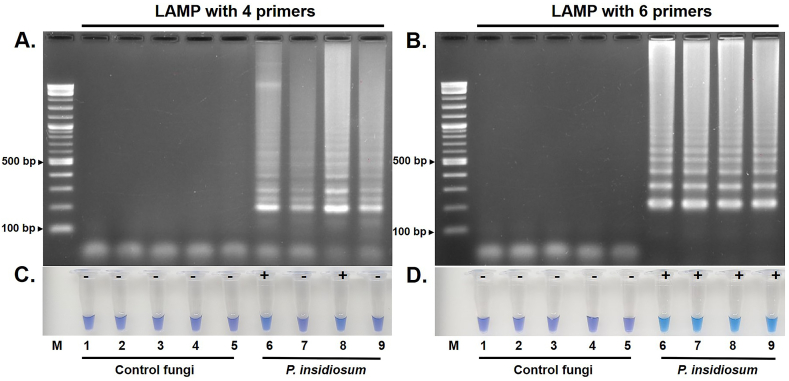
**c-LAMP using 4 and 6 *P. insidiosum*-targeted primers.** DNA samples from control fungi (Numeric labels 1–5 represent *A. fumigatus*, *Neoscytalidium* spp., *A. glaucus*, *A. terreus*, and *P. variotii*, respectively) and *P. insidiosum* (Numeric labels 6–9) are assessed by the c-LAMP assay using (A and C) 4 primers (F3, B3, FIP, and BIP) and (B and D) 6 primers (F3, B3, FIP, BIP, LF, and LB). Amplicons are detected by (A and B) agarose gel electrophoresis and (C and D) HNB colorimetric method. "M" depicts the DNA ladder markers. "+" indicates a positive c-LAMP reaction tube (sky blue), while "-" indicates a negative one (violet), as determined by the HNB colorimetric method.

Representative DNA samples from *P. insidiosum* (*n* = 4) and various fungi (*n* = 5) were used for assay optimization. HNB was added to the regular LAMP reaction using a set of 4 *P. insidiosum*-targeted primers (F3, B3, FIP, and BIP) [17]. The agarose gel electrophoresis showed the typical positive sign of ladder-like amplicons for all *P. insidiosum* samples but not for the fungi (Fig. 1A). The colorimetric method, employing HNB color readout by the naked eye, only detected 2 positive samples from *P. insidiosum*, as the dye color changed from violet to sky blue in the reaction tube, while the other DNA samples (2 *P. insidiosum* and 5 fungi) were determined negative, as the reaction tubes remained violet (Fig. 1C).

Upon adding 2 more *P. insidiosum*-targeted loop primers (LF and LB) in the regular LAMP reaction, the agarose gel electrophoresis showed relatively more robust LAMP products for all *P. insidiosum* samples, while no bands were observed for the control fungi (Fig. 1B). For the HNB colorimetric method, all *P. insidiosum* samples were determined positive (the reaction tubes turned sky blue), while all control fungi were determined negative (the color of the reaction tubes was unchanged) (Fig. 1D).

### 3.2 The detection limit of c-LAMP

**Fig. 2:**
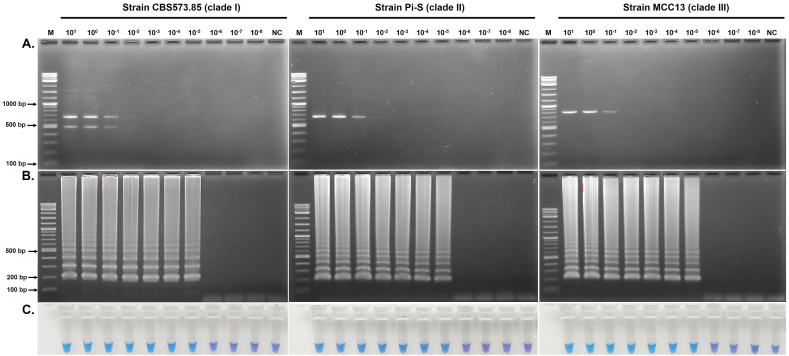
**Detection limits of c-LAMP and m-PCR for *P. insidiosum* detection.** DNA samples are from 3 *P. insidiosum* strains: CBS573.85 (clade I), Pi-S (clade II), and MCC13 (clade III). DNA templates (10-fold dilution) range from 10 to 1 x 10^−8^ ng per reaction, as indicated in the picture, for testing with m-PCR (A) and c-LAMP (B and C) assays. The result-reading methods include agarose gel electrophoresis (A and B) and HNB colorimetric method (C). "NC" represents the no-template control, while "M" depicts the DNA ladder markers. "+" indicates a positive c-LAMP reaction tube (sky blue), while "-" indicates a negative one (violet), as determined by the HNB colorimetric method.

DNA samples from 3 *P. insidiosum* strains (CBS573.85, Pi-S, and MCC13) were prepared to test c-LAMP's analytical sensitivity (Limit of Detection, or LOD). The goal was to determine the smallest amount of DNA that still produces a positive LAMP reaction and compare it with m-PCR. The tested DNA quantities (prepared in 10-fold dilution) ranged from 10 to 1 x 10^−8^ ng per reaction. c-LAMP consistently detected the lowest DNA amounts of 1 x 10^−5^ ng for all 3 *P. insidiosum* samples, regardless of the result-reading method used: gel electrophoresis (Fig. 2B) and colorimetric analysis (Fig. 2C). In comparison, m-PCR was able to detect *P. insidiosum* DNA in all samples at amounts as low as 0.1 ng while accurately identifying the organism's genotypes based on the presence of specific bands (490-bp and 660-bp for clade I, 660-bp for clade II, and 800-bp for clade III) (Fig. 2A).

### 3.3 Diagnostic performance of c-LAMP in comparison with m-PCR

**Fig. 3:**
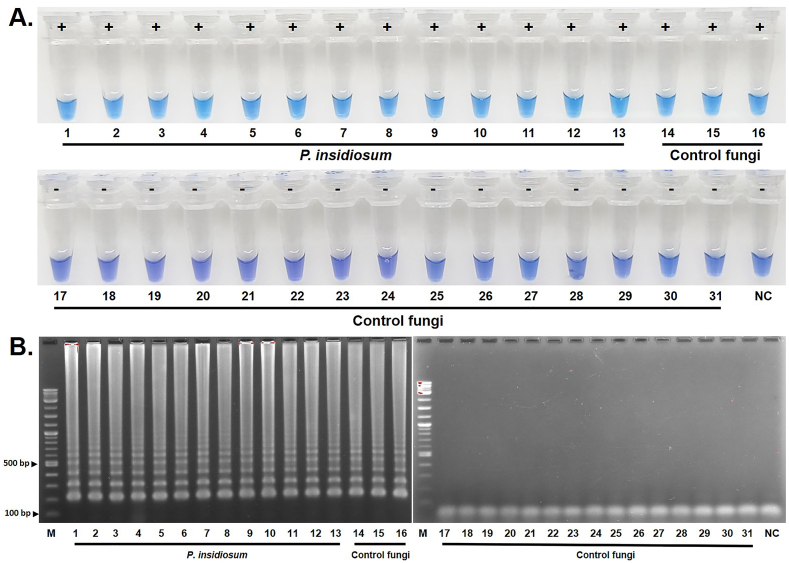
**Diagnostic performance of c-LAMP**. c-LAMP is evaluated for its diagnostic performance using DNA samples from various *P. insidiosum* isolates and control fungi. The results are obtained using (A) HNB colorimetric method and (B) gel electrophoresis. Numeric labels 1–13 are representative DNA samples from *P. insidiosum* strains CBS 578.85, CBS 579.85, EQ (05), EQ (09), CBS101039, CM07, P40, KCB06, P13, P26, P33, P49, and KCB08, respectively. Numeric labels 14–31 represent DNA samples from various control fungi (i.e., *P. aphanidermatum*, *P. catenulatum*, *P. rhizo-oryzae*, *Alternaria* spp., *A. fumigatus*, *A. flavus*, *Cladosporium* spp., *Curvularia* spp., *Daldinia* spp., *E. rostratum*, *Fusarium* spp., *H. capsulatum*, *M. gypseum*, *P. variotii*, *Penicillium* spp., *Syncephalastrum* spp., *Trichoderma* spp., and *T. rubrum*, respectively). "M" depicts the DNA ladder markers. "NC" is the no-template control. "+" indicates a positive c-LAMP reaction tube (sky blue), while "-" indicates a negative one (violet), as determined by the HNB colorimetric method.

Both c-LAMP and m-PCR assays detected all 51 *P. insidiosum* samples (Table 1). c-LAMP did not produce an amplicon from most control samples, except for *Pythium aphanidermatum*, *Pythium catenulatum*, and *Pythium rhizo-oryzae* (samples 14–16 shown in Fig. 3A and B). In contrast, m-PCR generated no product from all control fungi tested (Table 1). c-LAMP demonstrated a detection sensitivity, specificity, and accuracy of 100.0 %, 95.7 %, and 97.5 %, respectively, while the m-PCR assay achieved 100.0 % for all three parameters (Table 2). Notably, c-LAMP using the HNB colorimetric readout method showed a significantly faster turnaround time from sample application to result report, taking only 65 min, as opposed to the 125 and 180 min required for regular LAMP and m-PCR, which necessitate gel electrophoresis for result readout (Table 2).

**Table 2:** Diagnostic performance of c-LAMP and m-PCR for detecting *P*. *insidiosum*.

Assay type	c-LAMP		m-PCR
Result readout method	Colorimetric method	Gel electrophoresis	Gel electrophoresis
**Sensitivity (% [95 % CI**^a^**])**	100.0 [93.0–100.0]	100.0 [93.0–100.0]	100.0 [93.0–100.0]
**Specificity (% [95 % CI])**	95.7 [87.9–99.1]	95.7 [87.9–99.1]	100.0 [94.8 − 100.0]
**Accuracy (% [95 % CI])**	97.5 [92.9–99.4]	97.5 [92.9–99.4]	100.0 [97.0–100.0]
**Turnaround time**^b^**(min)**	65	125	180

a Confidence interval.

b Assay duration from DNA sample application to result readout.

### 3.4 Possible cause of the c-LAMP false positive results

DNA carry-over contamination in the laboratory environment was explored as a possible cause of the c-LAMP false positive results from the *P. aphanidermatum*, *P. catenulatum*, and *P. rhizo-oryzae* samples. After chemical reagents, plastic wares (i.e., pipette tips and tubes), and other related supplies were changed to a new clean set, followed by laboratory decontaminating and relocating the working space, c-LAMP against all false positive samples were repeated. As a result, false positives for these samples persisted.

**Fig. 4:**
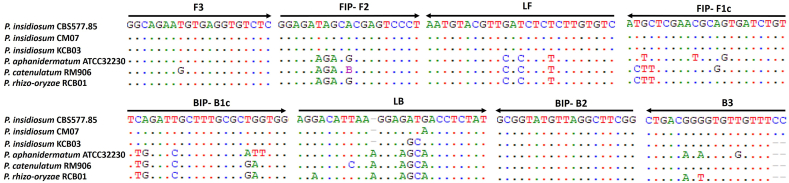
**Multiple alignments of LAMP primers and target ITS sequences**. Each long-tailed arrow represents a LAMP primer (F3, B3, FIP, BIP, LF, or LB) in the 5′-to-3′ direction at its corresponding target ITS sequences from 3 *P. insidiosum* strains, and one each of *P. aphanidermatum*, *P. catenulatum*, and *P. rhizo-oryzae*. The letters A, T, C, and G represent nucleotides, while the dots show that the aligned nucleotides from different organisms are identical. F3, B3, FIP, BIP, LF, and LB are forward outer primer, backward outer primer, forward inner primer (consisting of an F2 region at the 3′-end and an F1c region at the 5′-end), backward inner primer (consisting of a B2 region at the 3′-end and a B1c region at the 5′-end), loop forward primer, and loop backward primer, respectively.

The LAMP primers were designed to target the specific ITS sequence of *P. insidiosum*. To examine the possibility of cross-priming with the DNA of the other *Pythium* species, the primer sequences and the target sequences (233-bp long) from 3 *P. insidiosum* strains (accessions: HQ643569.1, EF016862.1, and LC556034.1), and one each of *P. aphanidermatum* (accession: LC550295.1), *P. catenulatum* (accession: LC556067.1), and *P. rhizo-oryzae* (accession: LC556053.1) were aligned (Fig. 4). Except for the B2 region of backward inner primer (BIP), most *P. insidiosum*-targeted LAMP primers can anneal the ITS sequences of all other *Pythium* species, with up to 6 nucleotide mismatches within the internal primer sequences. Additionally, no mismatch was observed at the 3′-end of any LAMP primers.

## 4 Discussion

This study aimed to develop a c-LAMP assay for detecting *P. insidiosum* using a one-step closed-tube process that allows instant visual identification of the resulting amplicons. Typically, a classical LAMP reaction requires 4 basic primers: 2 outer primers (F3 and B3) and 2 inner primers (FIP and BIP). These primers are sufficient for LAMP amplification, as confirmed by agarose gel analysis [13,15]. In this study, the color indicator dye, HNB, was added to our previously established LAMP reaction using 4 inner/outer primers (F3, B3, FIP, and BIP) [17] to test the representative DNA samples from *P. insidiosum* and control organisms. The initial assessment against 5 different fungi showed that the LAMP assay was specific, as all these control samples were determined negative by gel electrophoresis (Fig. 1A) and HNB colorimetric method (Fig. 1C). However, while all *P. insidiosum* samples were determined positive using the gel electrophoresis (Fig. 1A), only 2 were read as positive using the colorimetric method (Fig. 1C). This observation highlights the critical need for assay optimization to enhance assay sensitivity. It has been known that using 6 primers (2 each of inner, outer, and loop primers) in LAMP assays significantly improves the amplification efficiency [[25], [26], [27], [28], [29], [30], [31], [32],^38^]. Our study supplemented the original LAMP reaction [17] with 2 loop primers (LF and LB). This modification resulted in a stronger amplification reaction, enabling the precise identification of all *P. insidiosum* samples checked by gel electrophoresis (Fig. 1B) and colorimetric method (Fig. 1D). Crucially, this adjustment did not affect the assay specificity, as all control fungi were tested negative. Therefore, including loop primers in the reaction is essential for successfully developing c-LAMP [[25], [26], [27], [28], [29], [30], [31], [32]]. The optimized c-LAMP using 6 primers was then used in the downstream analytic sensitivity and diagnostic performance assessments.

The analytical sensitivity of c-LAMP for detecting *P. insidiosum* DNA, compared to m-PCR, was systematically assessed in this study (Fig. 2). The template DNA samples, which ranged from 10 ng to 1 x 10^−8^ ng, were prepared through a 10-fold serial dilution. Notably, the LOD analysis may face experimental errors when reporting analytical sensitivity lower than 1 ng. However, each amplification reaction was performed 3 times, and the results were verified by 2 independent researchers to ensure consistency. Our results indicate that c-LAMP can identify minimal quantities of *P. insidiosum* DNA, with LOD reaching as low as 1 x 10^−5^ ng across all three tested strains. Such the low LOD for c-LAMP was equivalently detected by gel electrophoresis (Fig. 2B) and colorimetric method (Fig. 2C). It is important to note that the HNB colorimetric method, in particular, is much more cost-effective and convenient than gel electrophoresis, as it does not require specialized equipment and can provide results in a markedly shorter time frame. Regarding m-PCR, the assay demonstrated a LOD of 0.1 ng (Fig. 2A), indicating its 10,000 times lower sensitivity than c-LAMP when detecting trace amounts of *P. insidiosum* DNA. Our findings were consistent with those of other investigators who report LAMP is more sensitive than conventional PCR [10,39,40]. However, m-PCR holds its advantage in accurately genotyping *P. insidiosum*, as it distinctly identified genotypes based on the differential band patterns. This genotyping capability is crucial for epidemiological studies, which c-LAMP lacks. Nevertheless, the striking analytical sensitivity underscores the potential of c-LAMP as a reliable method for early *P. insidiosum* detection, even in samples where the pathogen's DNA is present in very low quantities.

To effectively differentiate between positive and negative results in the c-LAMP assay, we recommend analyzing unknown samples alongside positive and negative controls each time the assay is performed. The color of the unknown sample should be compared to the observed colors of the positive control (sky blue) and the negative control (violet) to accurately interpret the c-LAMP results. Based on the LOD findings of our c-LAMP assay, the observed color can reliably indicate the presence of template DNA at concentrations as low as 1 x 10^−5^ ng (Fig. 2). This demonstrates that color-based interpretation is effective even with minimal template DNA. However, if there is any uncertainty regarding color interpretation, especially with samples containing very low levels of target DNA, the result should be verified by examining the ladder-like pattern of the c-LAMP product using conventional agarose gel electrophoresis.

c-LAMP demonstrated 100 % detection sensitivity in identifying all *P. insidiosum* samples (Table 1, Table 2; Fig. 3). This finding reflects the assay's strength and potential as a reliable screening tool for this pathogen. However, c-LAMP generated amplicons for a few control samples (*P. aphanidermatum*, *P. catenulatum*, and *P. rhizo-oryzae*), resulting in a detection specificity of 95.7 % (Table 1, Table 2; Fig. 3). This cross-reactivity suggests further refinement to improve the assay's performance. Despite this limitation, the assay maintained an overall accuracy of 97.5 %, making it highly reliable for identifying *P. insidiosum* in a given sample. On the other hand, m-PCR displayed 100.0 % specificity and sensitivity, accurately detecting all *P. insidiosum* samples and producing no product from all control fungi. The extreme specificity of m-PCR makes it a valuable tool for confirming the presence of *P. insidiosum* in samples. When considering these assays, turnaround time is an important factor. c-LAMP provided results in 65 min, surpassing the m-PCR assay, which took up to 180 min. Another advantage of c-LAMP is its simplicity, with results obtainable through direct visual inspection using colorimetric analysis. This feature makes c-LAMP an appealing assay for routine diagnostics, especially in resource-limited settings where access to advanced laboratory equipment (i.e., thermocycler and gel electrophoresis apparatus) may be limited [25,26,29,30].

LAMP assays, known for their high sensitivity, can sometimes yield false-positive results [41,42]. Factors contributing to these false positives include the formation of primer dimers, primer cross-priming, cross-contamination between samples, and carry-over contamination from previously amplified DNA products [41,[43], [44], [45], [46]]. In our current study, we identified 3 false positive results using c-LAMP in samples from closely related *Pythium* species: *P. aphanidermatum*, *P. catenulatum*, and *P. rhizo-oryzae*. These false positives might have resulted from carry-over contamination of previously amplified *P. insidiosum* DNA in the laboratory. Our efforts to fix this issue included replacing chemical supplies and containers, laboratory decontaminating, and moving the working space, yet the false positives for these samples persisted. Another potential cause might relate to the primers used in c-LAMP, which were designed to target the specific ITS sequence. Such primers may also bind to similar regions in the DNA of the 3 aforementioned *Pythium* species. This phenomenon, known as cross-priming, was explored by aligning the primer target sequences (233-bp long) from 3 *P. insidiosum* strains and the comparable sequences from *P. aphanidermatum*, *P. catenulatum*, and *P. rhizo-oryzae*. The analysis revealed that the LAMP primers intended for *P. insidiosum* could also bind to the DNA of the 3 other *Pythium* species. Despite noticeable differences in the nucleotide sequences where the primers anneal, the efficiency of c-LAMP was not affected for these non-target species. Thus, c-LAMP is surprisingly tolerant of differences between the primers and their target sequences, although the reasons for this are not fully understood [46]. To possibly resolve the issue of primer-target sequence tolerance, adjusting the nucleotide matching at the 3′-end of the primers, crucial for the initial sequence extension, was considered. However, this was not feasible in our case due to the limited length and variability of the target sequences. Despite this challenge, these closely related *Pythium* species in samples could not be considered clinically significant, as no infections in humans or animals have been reported for *P. catenulatum* and *P. rhizo-oryzae*. Besides, only 3 cases of *P. aphanidermatum* infection have been documented, indicating a very low incidence rate of this condition [[47], [48], [49]].

In conclusion, the newly developed closed-tube c-LAMP assay offers a rapid, cost-effective, and efficient method for detecting *P. insidiosum*. Utilizing HNB dye, the assay provides easy-to-interpret results through a color change from violet to sky blue, facilitating detection without the need for expensive laboratory equipment like gel electrophoresis apparatus and thermocyclers. The c-LAMP reaction requires only a single optimal temperature, making a simple heat block set at 65 °C sufficient to perform the assay. Demonstrating a 100.0 % sensitivity, 95.7 % specificity, and 97.5 % accuracy with a detection limit significantly lower than that of the reference m-PCR assay, c-LAMP proved to be a promising diagnostic tool, especially suitable for use in resource-constrained environments where advance equipment is lacking, and pythiosis is prevalent. With its capability for fast turnaround, c-LAMP significantly outperformed traditional molecular methods, requiring only 65 min to deliver results, offering a practical solution to address urgent diagnostic needs in remote areas.

## CRediT authorship contribution statement

**Thanawat Sridapan:** Writing – review & editing, Writing – original draft, Visualization, Methodology, Conceptualization. **Chalisa Jaturapaktrarak:** Writing – review & editing, Resources, Methodology. **Thidarat Rujirawat:** Writing – review & editing, Visualization, Resources. **Atisak Jiaranaikulwanich:** Writing – review & editing, Resources, Methodology. **Chompoonek Yurayart:** Writing – review & editing, Resources, Methodology. **Theerapong Krajaejun:** Conceptualization.

## Informed consent statement

Not applicable.

## Institutional review board statement

Not applicable (no humans and animals are involved).

## Data availability statement

Not applicable.

## Declaration of generative AI and AI-assisted technologies in the writing process

During the preparation of this work, the authors used Grammarly software to enhance the grammar and improve the readability of this manuscript. After using this tool/service, the authors reviewed and edited the content as needed and take full responsibility for the content of the publication.

## Funding

This work was supported by the 10.13039/501100004704National Research Council of Thailand and 10.13039/501100004156Mahidol University, Thailand (Grant numbers: N42A650339; T.K.) and Faculty of Medicine, Ramathibodi Hospital, 10.13039/501100004156Mahidol University, Thailand (Grant number: CF_67004; T.K.).

## Declaration of competing interest

The authors declare the following financial interests/personal relationships which may be considered as potential competing interests:Theerapong Krajaejun reports financial support was provided by 10.13039/501100004704National Research Council of Thailand and 10.13039/501100004156Mahidol University. Theerapong Krajaejun has patent pending to Mahidol University. If there are other authors, they declare that they have no known competing financial interests or personal relationships that could have appeared to influence the work reported in this paper.
